# Enhancive effects of Lewis y antigen on CD44-mediated adhesion and spreading of human ovarian cancer cell line RMG-I

**DOI:** 10.1186/1756-9966-30-15

**Published:** 2011-02-07

**Authors:** Lili Gao, Limei Yan, Bei Lin, Jian Gao, Xiuyun Liang, Yanyan Wang, Juanjuan Liu, Shulan Zhang, Iwamori Masao

**Affiliations:** 1 Department of Obstetrics and Gynecology, Shengjing Hospital Affiliated to China Medical University, Shenyang, 110004, P R of China; 2 Departments of Biochemistry, Faculty of Science and Technology, Kinki University, Osaka, 577-8502, Japan

## Abstract

**Background:**

This study aimed to investigate the molecular structural relationship between cell adhesive molecule CD44 and Lewis y antigen, and determine the effects of Lewis y antigen on CD44-mediated adhesion and spreading of ovarian cancer cell line RMG-I and the Lewis y antigen-overexpressed cell line RMG-I-H.

**Methods:**

The expression of CD44 in RMG-I and RMG-I-H cells before and after treatment of Lewis y monoclonal antibody was detected by immunocytochemistry; the expression of Lewis y antigen and CD44 was detected by Western Blot. The structural relationship between Lewis y antigen and CD44 was determined by immunoprecipitation and confocal laser scanning microscopy. The adhesion and spreading of RMG-I and RMG-I-H cells on hyaluronic acid (HA) were observed. The expression of CD44 mRNA in RMG-I and RMG-I-H cells was detected by real-time RT-PCR.

**Results:**

Immunocytochemistry revealed that the expression of CD44 was significantly higher in RMG-I-H cells than in RMG-I cells (*P *< 0.01), and its expression in both cell lines was significantly decreased after treatment of Lewis y monoclonal antibody (both *P *< 0.01). Western Blot confirmed that the content of CD44 in RMG-I-H cells was 1.46 times of that in RMG-I cells. The co-location of Lewis y antigen and CD44 was confirmed by co-immunoprecipitation. The co-expression of CD44 and Lewis y antigen in RMG-I-H cells was 2.24 times of that in RMG-I cells. The adhesion and spreading of RMG-I-H cells on HA were significantly enhanced as compared to those of RMG-I cells (*P *< 0.01), and this enhancement was inhibited by Lewis y monoclonal antibody (*P *< 0.01). The mRNA level of CD44 in both cell lines was similar (*P *> 0.05).

**Conclusion:**

Lewis y antigen strengthens CD44-mediated adhesion and spreading of ovarian cancer cells.

## Background

Glycosylated antigens, important components of glycolipids and glycoproteins, are widely expressed on cell membrane and are involved in cell adhesion, recognition, and signal transduction [1]. The alterations of type II sugar chains, such as Lewis × and Lewis y, are common in ovarian cancer: 75% of epithelial ovarian cancers have overexpression of Lewis y antigen which shows obvious relationship with prognosis; tumor marker CA125 in epithelial ovarian cancer also contains Lewis y structure [2,3]. Alpha1, 2-fucosyltransferase (α1, 2-FT) is a key enzyme for synthesizing Lewis y antigen. In our previous study, we successfully transferred α1, 2-FT gene into ovarian cancer cell line RMG-I and established a cell line RMG-I-H with stable high expression of Lewis y antigen, which showed obviously enhanced malignant behaviors [4-6].

CD44, one of important adhesive molecules on cells, is involved in the adhesion and metastasis of tumor cells and plays an important role in tumor development [7-10], but the regulatory mechanism is unclear yet. The molecule CD44 is abundant of α-L-fucose, and is an important α1, 2-fucose antigen-containing protein on the surface of cells [11]. CD44 is expressed on several tissue cells, binds to receptors in extracellular matrix such as hyaluronic acid (HA) and laminin, and mediates cell-cell and cell-matrix adhesion [12,13]. The present study aimed to determine the impact of α1, 2-FT gene transfection on the expression of CD44 on cells and the effects of Lewis y antigen on CD44-mediated cell adhesion and spreading.

## Methods

### Materials

Lewis y monoclonal antibody was purchased from Abcam Co.; CD44 monoclonal antibody from Santa Cruz Co. and Wuhan Boster Co.; Protein A-agarose, ECL chromogenic agent, and 5× SDS-PAGE loading buffer from Shanghai Beyotime Institute of Biotechnology; SABC kit from Beijing Zhongshan Golden Bridge Biotechnology Co., Ltd; HA from Hefei Bomei Biotechnology Co., Ltd; DMEM culture medium from Gibco Co.; fetal bovine serum (FBS) from Shenyang Boermei Reagent Co.; Coomassie brilliant blue from Beijing Solarbio Science & Technology Co., Ltd; Trizol reagent, PrimeScript™RT reagent kit, and SYBR^® ^Premix Ex Taq™from Dalian TaKaRa Biotechnology Co. The sequences of primers were synthesized by Shanghai Invitrogen Co.

### Cell line and cell culture

The cell line RMG-I was originated from ovarian clear cell cancer tissues. The cell line RMG-I-H with high expression of α1, 2-FT and Lewis y antigen was established in our lab [14]. RMG-I and RMG-I-H cells were cultured in DMEM medium containing 10% FBS at 37°C in 5% CO_2 _and saturated humidity. Cells are grouped in immunocytochemistry, cell spreading, cell adhesion as follows: negative groups, Lewis y antibody-untreated groups, Lewis y antibody-treated groups (single layer cells were treated with 10 μg/mL Lewis y monoclonal antibody at 37°C in 5% CO_2 _for 60 min), irrelevant isotype-matched control(10 μg/mL normal mouse IgM).

### Immunocytochemistry

RMG-I-H and RMG-I cells at exponential phase of growth were digested by 0.25% trypsin and cultured in DMEM medium containing 10% FBS to prepare single-cell suspension. Cells were washed twice with cold PBS when growing in a single layer, and fixed with 4% paraformaldehyde for 30 min. The expression of CD44 on cells was detected according to the SABC kit instructions. The concentration of CD44 monoclonal antibody was 1:100. The primary antibody was replaced by PBS for negative control. 10 μg/mL normal mice IgM acted as irrelevant isotype-matched control. The average optical densities were measured under a microscope with image processing, being presented as the means ± standard deviation for three separate experiments.

### Confocal laser scanning microscopy

After fixing with 4% paraformaldehyde, RMG-I-H cells were treated by the one-step immunofluorescence dual-labeling method. In brief, mouse anti-human Lewis y antibody and rabbit anti-human CD44 antibody were diluted to 1:100 as primary antibody solutions; goat anti-rabbit TRITC-labeled secondary antibody and goat anti-mouse FITC-labeled secondary antibody were diluted to 1:200. Cells were blocked by normal goat serum for 30 min, added with primary antibody solutions at 37°C for 1 h, then cultured at room temperature overnight. After washing with PBS, cells were added with secondary antibody solutions at 37°C for 1 h, stained with 4, 6-diamidino-2-phenylindole (PI) for 5 min, then observed under the confocal laser scanning microscope. The data were colleted by a computer for digital imaging. The experiment was repeated 3 times.

### Western Blot

RMG-I-H and RMG-I cells at exponential phase of growth were washed twice with cold PBS, added with cell lysis buffer (0.2 mL/bottle), placed on ice for 15 min, then centrifuged at 14,000 rpm for 15 min. The protein concentration in the supernatant was detected by the method of Coomassie brilliant blue. The supernatant was cultured with 1× SDS-PAGE loading buffer at 100°C for 5 min for protein denaturation. Then, 50 μg of the protein was used for SDS-PAGE gel electrophoresis. The protein was transferred onto PVDF membrane, blocked by 5% fat-free milk powder at room temperature for 2 h, added with primary mouse anti-human CD44 monoclonal antibody (1:200) and mouse anti-human Lewis y monoclonal antibody (1:1000) and cultured at 4°C overnight, then added with secondary HRP-labeled goat anti-mouse IgG (1:5000) and cultured at room temperature for 2 h, and finally visualized by ECL reagent. The experiment was repeated 3 times.

### Co-immunoprecipitation

The protein was extracted from cells before and after transfection with the method described in *Western Blot *section. After protein quantification, 500 μg of each cell lysis was added with 1 μg of CD44 monoclonal antibody and shaken at 4°C overnight, then added with 40 μL of Protein A-agarose and shaken at 4°C for 2 h, finally centrifuged at 2500 rpm for 5 min and washed to collect the precipitation. The precipitated protein was added with 20 μL of 1× SDS-PAGE loading buffer at 100°C for 5 min for denaturation. The supernatant was subjected to SDS-PAGE gel electrophoresis. Lewis y monoclonal antibody (1:1000) was used to detect Lewis y antigen. Other steps were the same as described in *Western Blot *section.

### Cell spreading

The 2 mg/mL HA-coated 35-mm culture dishes were placed at 37°C for 1 h, and then blocked by 1% bovine serum albumin (BSA) for 1 h. The single-cell suspension (15,000/mL) prepared with serum-free DMEM was added to the dishes (1 mL/well) and cultured at 37°C in 5% CO_2 _for 90 min. Under the inverted microscope, 3 to 5 visual fields (×200) were randomly selected to count 200 cells: the round and bright cells were counted as non-spreading cells; the oval cells with pseudopods were counted as spreading cells. Irrelevant control antibodies (10 mg/ml) are used to evaluate the specificity of the inhibitions. The experiment was repeated 3 times.

### Cell adhesion

The 96-well plates were coated with 2 mg/ml HA (50 μL/well). The plate coated with 3 mg/mL polylysine and 1% BSA was used as maximal and minimal adhesion controls, respectively. After 2-hour coating at 37°C, the plates were washed twice with PBS, and blocked again with 1% BSA for 2 h. The cells were digested by 0.25% trypsin, centrifuged at 1000 rpm for 5 min, and then added with serum-free DMEM culture medium to prepare single-cell suspension. Cells were diluted to 5 × 10^4^/mL, added to coated plates (100 μL/well) and cultured at 37°C in 5% CO_2 _for 2 h. After washing off the un-adhered cells, the 96-well plates were fixed by 4% paraformaldehyde for 30 min, stained with 0.5% crystal violet (100 μL/well) for 2 h, and then washed twice with cold PBS. The absorbance at 597 nm (*A*_597 _absorbance represents the adhesive cells) was detected by a microplate reader. Irrelevant control antibodies (10 mg/ml) are used to evaluate the specificity of the inhibitions. The experiment was repeated 3 times.

### Detecting CD44 mRNA in RMG-I and RMG-I-H cells by real-time PCR

RMG-I and RMG-I-H cells at exponential phase of growth were added with Trizol reagent (1 mL per 1 × 10^7 ^cells) to extract total RNA. The concentration and purity of RNA were detected by an ultraviolet spectrometer. cDNA was synthesized according to the RNA reverse transcription kit instructions (TaKaRa Co.). The reaction system contained 4 µL of 5× PrimeScript™Buffer, 1 µL of PrimeScript™RT Enzyme Mix I, 1 µL of 50 µmol/L Oligo dT Primer, 1 µL of 100 µmol/L Random 6 mers, 2 µL of total RNA, and 11 µL of RNase-free dH_2_O. The reaction conditions were 37°C for 15 min, 85°C for 5 s, and 4°C for 5 min. The sequences of CD44 gene primers were 5'-CCAATGCCTTTGATGGACCA-3' for forward primer and 5'-TGTGAGTGTCCATCTGATTC-3' for reverse primer. The sequences of α1,2-FT gene primers were 5'-AGGTCATCCCTGAGCTGAAACGG-3' for forward primer and 5'-CGCCTGCTTCACCACCTTCTTG-3' for reverse primer. The sequences of β-actin gene primers were 5'-GGACTTCGAGCAAGAGATGG-3' for forward primer and 5'-ACATCTGCTGGAAGGTGGAC-3' for reverse primer. The reaction system for real-time fluorescent PCR contained 5 µL of 2× SYBR^® ^Premix Ex Taq™, 0.5 μL of 5 μmol/L PCR forward primer, 0.5 μL of 5 μmol/L PCR reverse primer, 1 µL of cDNA, and 3 µL of dH_2_O. The reaction conditions were 45 cycles of denaturation at 95°C for 20 s and annealing at 60°C for 60 s. The Light Cycler PCR system (Roche Diagnostics, Mannheim, Germany) was used for real-time PCR amplification and Ct value detection. The melting curves were analyzed after amplification. PCR reactions of each sample were done in triplicate. Data were analyzed through the comparative threshold cycle (CT) method.

### Statistical analyses

All data are expressed as mean ± standard deviation and were processed by the SPSS17.0 software. Raw data were analyzed by the variance analysis. A value of *P *< 0.05 was considered to be statistically significant.

## Results

### The expression of CD44 in RMG-I and RMG-I-H cells

**Figure 1:**
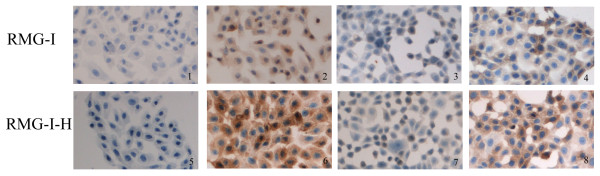
**The expression of CD44 in RMG-I and RMG-I-H cells detected by immunocytochemistry (×400)**. Panels 1 and 5 are negative controls; panels 2 and 6 are Lewis y antibody-untreated cells; panels 3 and 7 are Lewis y antibody-treated cells; panels 4 and 8 are cells treated by irrelevant isotype-matched control. The expression of CD44 was detected by SABC methods in RMG-I and RMG-I-H cells, and brown color degree by DAB staining indicated the expression level of CD44. It can be seen from the figure that the expression of CD44 in the RMG-I-H cells was stronger than that in RMG-I cells, which was decreased after Lewis y antibody blocking.

Immunocytochemistry showed that the positive CD44 staining presented as light yellow particles in the cytoplasm of RMG-I cells and brown-yellow particles in the cytoplasm and on the membrane of RMG-I-H cells (Figure 1). The relative level of CD44 expression was significantly higher in RMG-I-H cells than in RMG-I cells (*P *< 0.01) (Table 1).

**Table 1:** The average optical density on immunocytochemical staining with CD44 antibodies.

Group	RMG-I	RMG-I-H
Negative control	0.02 ± 0.03	0.03 ± 0.01

Lewis y antibody-untreated	0.28 ± 0.02	0.49 ± 0.02*

Lewis y antibody-treated	0.11 ± 0.01**	0.11 ± 0.01**

Irrelevant isotype-matched control	0.26 ± 0.01	0.46 ± 0.01

* *P *< 0.01, vs. RMG-I cells; ** *P *< 0.01, vs. Irrelevant isotype-matched control.

After treatment of Lewis y monoclonal antibody, the expression of CD44 was decreased in both RMG-I-H cells and RMG-I cells (*P *< 0.01), moreover showed no significant difference between the two cell lines (*P *> 0.05); after treatment of normal mouse IgM, the expression of CD44 did not change in RMG-I-H cells and RMG-I cells, compared with Lewis y antibody-untreated groups(Figure 1 Table 1).

### Co-location of CD44 and Lewis y antigen on RMG-I-H cells

**Figure 2:**
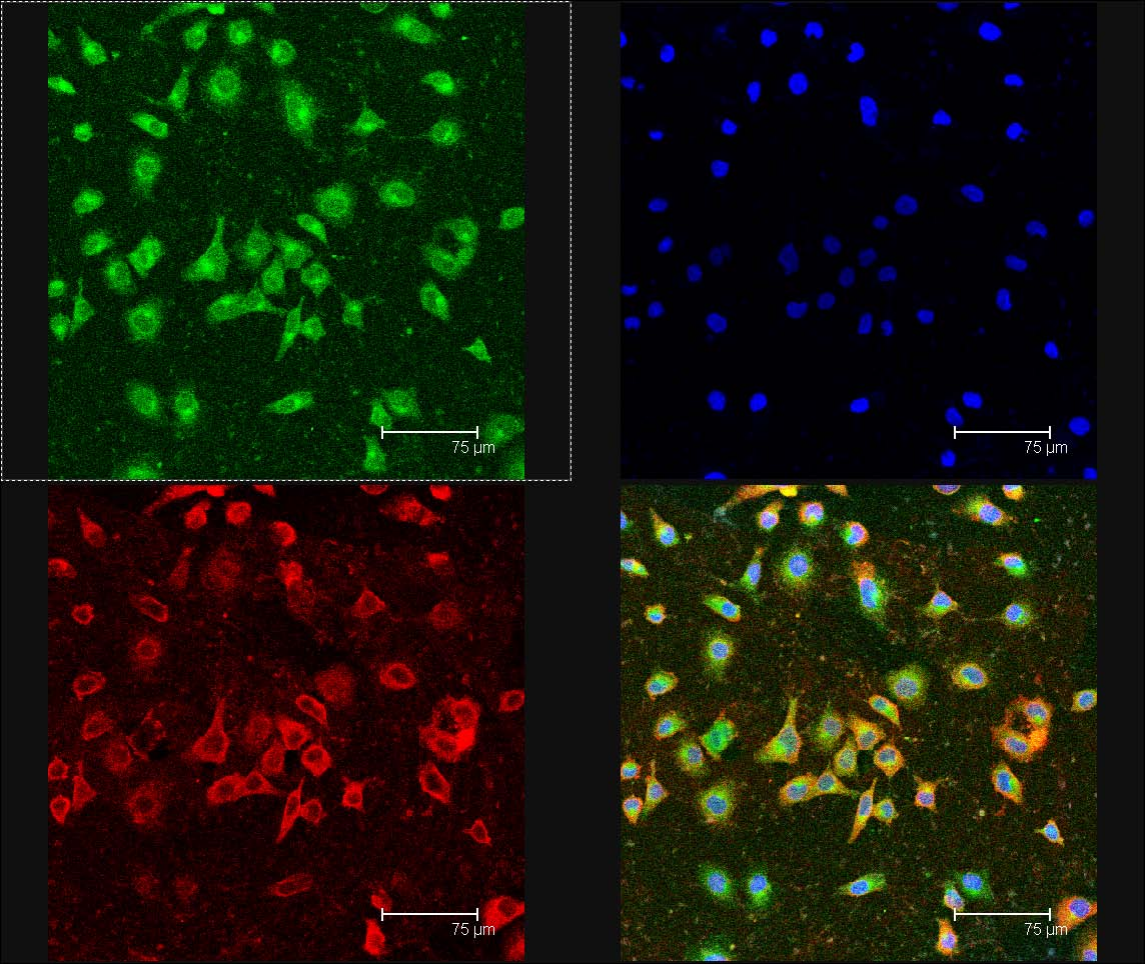
**Co-location of CD44 and Lewis y antigen on RMG-I-H cells observed under confocal laser scanning microscope**. Red fluoscence on the upper left panel indicates CD44 expression; green fluoscence on the upper right panel indicates Lewis y antigen expression; blue fluoscence on the upper right panel indicates cell nuclear location; the lower right panel is a merged image of the other three panels. Lewis y antigen CD44 mainly expressed in the cell membrane observed under the confocal laser scanning microscope, and it were seen as yellow fluorescence after the two overlap, suggesting that Lewis y antigen and CD44 co-localizated in the cell membrane.

Under the confocal laser scanning microscope, CD44 presented red fluoscence mainly on cell membrane and partly in cytoplasm; Lewis y antigen presented green fluoscence mainly on cell membrane (Figure 2). Both red fluoscence and green fluoscence were accumulated at the margin of cell clusters and overlapped as yellow fluoscence, indicating the co-location of CD44 and Lewis y antigen.

### The expression of CD44 and Lewis y antigen in RMG-I and RMG-I-H cells

**Figure 3:**
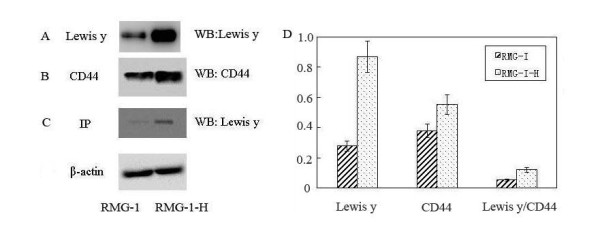
**The expression of CD44 and Lewis y antigen in RMG-I and RMG-I-H cells**. Panel A shows the expression of Lewis y antigen in RMG-I-H cells was higher than that in RMG-I; panel B shows the expression of CD44 in RMG-I-H cells was higher than that in RMG-I; panel C shows that Lewis y antigen, which in RMG-I-H cells was higher than that in RMG-I, was expressed both in RMG-I and RMG-I-H cells after CD44 immunoprecipitation; panel D Quantitative data were expressed as the intensity ratio target genes to beta-actin. (*P *< 0.01)

Western Blot showed that the expression of CD44 in RMG-I-H cells was significantly increased by 1.46 times of that in RMG-I cells (*P *< 0.01) (Figure 3.BD), and the expression of Lewis y antigen was significantly increased by 2.98 times (*P *< 0.01) (Figure 3.AD). Immunoprecipitation showed that, using the ratio of Lewis y antigen expression to CD44 expression to represent the relative expression of Lewis y antigen in CD44, the expression of Lewis y antigen in RMG-I-H cells was increased by 2.24 times of that in RMG-I cells (*P *< 0.01) (Figure 3.CD).

### The mRNA levels of CD44 and α1,2-FT in RMG-I and RMG-I-H cells

**Figure 4:**
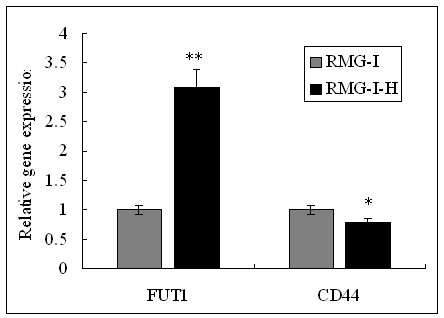
**The mRNA expression of CD44 and α1, 2-FT in RMG-I and RMG-I-H cells were tested by quantitative Real-Time RT-PCR**. The mRNA level of α1, 2-FT was significantly increased, but the mRNA level of CD44 was almost the same in RMG-1-hFUT cells and RMG-1 cells. (**P < 0.01, * *P *> 0.05).

The 2^-ΔΔCT ^value of mRNA level of CD44 in RMG-I-H cells is 79% of that in RMG-I cells, which had no significant difference (*P *> 0.05), whereas the mRNA level of α1,2-FT in RMG-I-H cells was increased by 3.07 times of that in RMG-I cells detected by Real-time PCR (*P *< 0.01). (Figure 4).

### HA-mediated cell adhesion and spreading

The adhesion of RMG-I-H cells to HA was significantly stronger than that of RMG-I cells (*P *< 0.01) (Table 2). The adhesion of RMG-I-H and RMG-I cells to HA after Lewis y antigen blocking was decreased respectively by 62.31% and 70.34% of irrelevant isotype-matched control (*P *< 0.01), and no difference was observed between these two cell lines (*P *> 0.05). Cell adhesion did not change after treatment of normal mouse IgM, compared with Lewis y antibody-untreated groups (*P *> 0.05).

**Table 2:** HA-mediated adhesion and spreading of RMG-I and RMG-I-H cells

	Cell adhesion		Cell spreading	
		
Group	RMG-I	RMG-I-H	RMG-I	RMG-I-H
Lewis y antibody-untreated	1.41 ± 0.20	2.57 ± 0.58*	34 ± 5	57 ± 6*

Lewis y antibody-treated	0.53 ± 0.03**	0.76 ± 0.27**	16 ± 5**	14 ± 4**

Irrelevant isotype-matched control	1.36 ± 0.15	2.44 ± 0.67	35 ± 6	59 ± 8

* *P *< 0.01, vs. RMG-I cells; ** *P *< 0.01, vs. Irrelevant isotype-matched control.

On HA-coated plates, spreading RMG-I-H cells were significantly more than spreading RMG-I cells (*P *< 0.01) (Table 2). Cell spreading showed similar changes as cell adhesion after Lewis y antigen blocking, suggesting that Lewis y antigen was involved in the interaction of CD44 and HA.

## Discussion

This article mainly found that Lewis y antigen, as a structure in CD44 molecule, strengthens CD44-mediated adhesion and spreading of ovarian cancer cells. Inhibiting the expression of CD44 or blocking its binding to receptors and downstream signal molecules can inhibit the progression of ovarian cancer.

Glycoconjugates, an important component of cell membrane, are involved in cell growth and differentiation [15]. Fucose, the terminal residue of synthesized sugar chains, is involved in constructing the sugar chain structure of some important growth factor receptors and plays an important role in tumorigenesis [16]. Studies showed that fucosylated antigens expressed in tumor cells are involved in several cellular functions and related to some malignant cell behaviors, including adhesion, recognition, and signal transduction, and that the increased fucosylated antigens benefit the invasion and migration of tumor cells [17,18]. Ovarian cancer mostly has changes of type II glycosylated antigens, such as Lewis x, Lewis y and H antigens, which mainly depend on the α1, 2-FT-catalyzed fucosylation of galactose residues at the non-reducing terminal [19]. Our previous study showed that ovarian cancer cell line RMG-I mainly expressed Lewis × antigen, and confirmed that the enhanced adhesion of Lewis × antigen-overexpressed cells to peritoneal mesothelia was weakened after Lewis × antigen blocking in nude mouse experiments, suggesting that Lewis × antigen is related to the intraperitoneal dissemination of RMG-I cells [20]. We transfected wild type α1,2-FT gene into ovarian cancer cell line RMG-I to establish the α1,2-FT-overexpressed cell line RMG-I-H, and found that the activity of α1,2-FT in RMG-I-H cells was enhanced by 20 to 30 times[5]. We also found that only Lewis × and Lewis y antigens in the type II lactose chain family were expressed, 42.6% of Lewis × antigen in RMG-I-H cells transformed into Lewis y antigen, and that the concentration of Lewis y antigen in RMG-I-H cells was increased by about 20 times of that in RMG-I cells[5]. After transfection of α1, 2-FT gene, while the expression of Lewis y antigen in RMG-I-H cells was increased, the malignant behaviors of cells were also enhanced, for examples, the G1 phase of meiosis was shortened, the colony formation rate on soft agar was increased, the growth of subcutaneous and intraperitoneal xenografts in nude mice was accelerated, and the drug-resistance was enhanced [6,21-23]. Lewis y antigen has dual fucosylations--one more fucose than Lewis × antigen. Lewis y monoclonal antibody or α-L-fucosidase can significantly inhibit the proliferation and adhesion of RMG-I-H cells [6,24], indicating that the effect of Lewis y antigen on cell behaviors is stronger that that of Lewis × antigen, which may due to the number of fucoses.

CD44, an important α1, 2-FT-containing protein on cell surface, is involved in the adhesion and metastasis of tumor cells, and plays an important role in tumor progression [9]. Our present study showed that after transfection of α1,2-FT gene, the expression of CD44 in RMG-I-H cells was significantly increased together with the increase of Lewis y antigen (*P *< 0.01). Confocal laser scanning microscopy confirmed the co-location of CD44 and Lewis y antigen, interpreted that Lewis y antigen was a structure in CD44. In 2010, Lin et al. [25] reported that both CD173(H2) and Lewis y(CD174) could immunoprecipitate with CD44 in breast cancer cells. Our results showed that the increase of Lewis y antigen was more obvious, which increased by 2.24 times after α1, 2-FT gene transfection (*P *< 0.05). Lewis y antibody can block the increase of CD44 expression. We used gene chip to detect the differential expression of genes in cells before and after transfection, and found that 88 genes were differentially expressed after transfection, which were involved in cell proliferation and adhesion, signal transduction, protein phosphorylation, transcription, apoptosis, and so on[22]. However, the change of CD44 after transfection was mainly at protein level, with no obvious change at mRNA level (*P *> 0.05). Yuan et al. [26] also believed that CD44 and its several subtypes have post-transcriptional modification, including the addition of glycosaminoglycan and glycosylation.

The functions of α1, 2-FT in CD44 molecule are unclear yet. Studies found that it can prevent decomposition by proteolytic enzyme, enhance cell-cell adhesion, and inhibit cell apoptosis [11]. Labarrière et al. [27] also found that CD44v6 in mouse colon cancer cells contains H antigen. Its fucose structure is involved in cell adhesion, and the increase of its expression is related to the decrease of the sensitivity to natural killer cells or the decrease of the cytotoxicity of lymphocyte-activated killer cells. Therefore, CD44v6 helps mouse colon cancer cells to escape from the recognition and killing by the immune system, prone to invade lymph nodes and form metastasis. Our study confirmed that the adhesion and spreading of RMG-I-H cells to HA in extracellular matrix were significantly enhanced (all *P *< 0.01). After Lewis y antigen blocked, the expression of CD44 in cells was decreased, cell adhesion and spreading were also significantly decreased (all *P *< 0.01), suggesting that Lewis y antigen plays an important role in mediating the adhesion of CD44 to HA in extracellular matrix. Yuan et al. [26] used α-L-fucosidase to treat breast cancer cells, and found that the expression of CD44 was decreased; the adhesion of tumor cells to matrix was decreased, resulting in a decrease of cell invasion. This finding confirms our deduction.

The interaction of CD44 and HA activates RhoA signals and Rho kinase, enhances serine/threonine phosphorylation on Gab-1 (Grb2-associated binder-1), induces PI3K activation, triggers the PI3K/Akt pathway, and is involved in the progression of breast cancer[28]. It is also confirmed that the binding of CD44 to HA induces c-Src kinase activation, and is involved in the metastasis of ovarian cancer cells by activating the c-Src kinase pathway [29]. Our previous study showed that the expression of Akt total protein in Lewis y antigen-overexpressed ovarian cancer cells did not change, but it phosphorylation was significantly enhanced; ZD1839 and Lewis y antibody decreased the level of phosphorylated Akt in Lewis y antigen-overexpressed cells, but showed no effect in the ovarian cancer cells with low Lewis y antigen expression. MTT assay showed that PI3K-specific inhibitor LY294002 can significantly inhibit the proliferation of Lewis y antigen-overexpressed ovarian cancer cells [30].

Ovarian cancer cells adhere to peritoneal mesothelia via the formation of several compounds: CD44/HA, β1-integrin/fibronectin, CA125/mesothelin, and so on [31,32]. HA and fibronectin are components of extracellular matrix. HA in extracellular matrix is a major ligand of CD44. Many studies proved the importance of CD44 and its receptors in the biological behaviors of ovarian cancer [33]. Studies found that oncostatin M and transforming growth factor 1 (TGF1) could mediate the binding of HA to CD44 in tumor cells originated from lung epithelia, leading to the glycosylation and phosphatization of CD44 [34]. CD44 and HA mediate the overexpression and activation of integrin as well as the adhesion of tumor cells to epithelia, and enhance the migration and metastasis of tumor cells [35]. Wielenga et al. [36] reported that, in colorectal cancer, heparin sulfate-modified CD44 showed increased ability of binding to hepatocyte growth factor/scatter factor (HGF/SF), thus presenting HGF/SF to c-Met and leading to c-Met phosphorylation, and triggering the c-Met signal pathway to activate lymphocyte function-associated antigen-1 (LFA-1), therefore, affecting the biological activities of tumor cells, such as angiogenesis and cell motivation. Zhang et al. [37] found that the binding of HA to CD44 affected the adhesion of tumor cells via some signal transduction pathways (such as the kinase C pathway), and played an important role in tumor metastasis. Kim et al. [38] used CD44 antibody to competitively inhibit the binding of HA to CD44, and found that the invasion of colorectal cancer cells to basement membranes was decreased by 95%. The above findings indicate that CD44 is involved in several signal transduction pathways related to tumor cell metastasis, and that inhibiting the expression of CD44 or blocking its binding to receptors can inhibit the metastasis of tumor cells. Our previous study showed that the expression of EGFR, TGF-βR, α5β1, and α5β3 was also increased in Lewis y antigen-overexpressed cells, and that Lewis y antigen, as an important structure in EGFR, TGF-βR, α5β1, and α5β3 (unpublished data), affected the biological behaviors of cells by activating the Raf/MEK/MAPK, PI3K/Akt, TGF-β/Smads, and FAK signal pathways[39,40].

In summary, Lewis y antigen is overexpressed on ovarian cancer cells, and is homogeneous in primary and metastatic lesions; hence, it has become a target antigen of immune therapy.

## Conclusions

We have transfected the alfa1, 2-fucosyltransferase gene into cultured cells from an ovarian carcinoma and showed that the transfected cells have elevated expression of CD44 with Lewis y resulting in their increased ability to adhere and to spread via the CD44-hyaluronic acid interaction. The paper demonstrates a novel role of Lewis y in regulating the CD44- hyaluronic interaction.

## Competing interests

The authors declare that they have no competing interests.

## Authors' contributions

LG carried out most parts of the experiment; LY, JG, XL, YW, JL and SZ participated in the experiment; BL participated in the design of the study; LY performed the statistical analysis; IM participated in its design and coordination and helped to draft the manuscript. All authors read and approved the final manuscript.

## References

[B1] Ugorski M Laskowska A Sialyl Lewis a: a tumor-associated carbohydrate antigen involved in adhesion and metastatic potential of cancer cells Acta Biochim Pol 2002 49 303 311 12362971

[B2] Diao B Lin B Lewis y antigen and its applications to tumor diagnosis and treatment J Modern Oncol 2009 17 132 134

[B3] Rodríguez-Burford C Barnes MN Berry W Partridge EE Grizzle WE Immunohistochemical expression of molecular markers in an avian model: a potential model for preclinical evaluation of agents for ovarian cancer chemoprevention Gynecol Oncol 2001 81 373 379 11371125 10.1006/gyno.2001.6191

[B4] Hao YY Lin B Zhao Y Zhang YH Li FF Diao B Ou YL Zhang SL α1, 2-Fucosyltransferase gene transfection influences on biological behavior of ovarian carcinoma-derived RMG-I cells Fen Zi Xi Bao Sheng Wu Xue Bao 2008 41 435 442 19137814

[B5] Iwamori M Tanaka K Kubushiro K Lin B Kiguchi K Ishiwata I Tsukazaki K Nozawa S Alterations in the glycolipid composition and cellular properties of ovarian carcinoma-derived RMG-I cells on transfecton of the alpha 1,2-fucosyltransferase gene Cancer Sci 2005 96 26 30 10.1111/j.1349-7006.2005.00005.x 15649251 PMC11158802

[B6] Li FF Lin B Hao YY Liu JJ Zhang F Zhang SL Inhibitory effect of anti-Lewis y antibody on α1,2-fucosyltransferase gene transfected human ovarian cancer cells in vitro Xi Bao Yu Fen Zi Mian Yi Xue Za Zhi 2008 24 267 269 18328190

[B7] Sy MS Mori H Liu D CD44 as a marker in human cancers Curr Opin Oncol 1997 9 108 112 10.1097/00001622-199701000-00017 9090502

[B8] Matsumura Y Tarin D Significance of CD44 gene products for cancer diagnosis and disease evaluation Lancet 1992 340 1053 1058 10.1016/0140-6736(92)93077-Z 1357452

[B9] Isacke CM Yarwood H The hyaluronan receptor, CD44 Int J Biochem Cell Biol 2002 34 718 721 10.1016/S1357-2725(01)00166-2 11950588

[B10] Alaniz L Cabrera PV Blanco G Ernst G Rimoldi G Alvarez E Hajos SE Interaction of CD44 with different forms of hyaluronic acid. Its role in adhesion and migration of tumor cells Cell Commun Adhes 2002 9 117 130 10.1080/15419060214522 12521133

[B11] Goupille C Marionneau S Bureau V Hallouin F Meichenin M Rocher J Le Pendu J α1,2-Fucosyltransferase increases resistance to apoptosis of rat colon carcinoma cells Glycobiology 2000 10 375 382 10.1093/glycob/10.4.375 10764825

[B12] Roa I Villaseca M Araya J Roa J de Aretxabala X Ibacache G García M CD44 (HCAM) expression in subserous gallbladder carcinoma J Rev Med Chil 2001 129 727 734 11552440

[B13] Murai T Miyazaki Y Nishinakamura H Sugahara KN Miyauchi T Sako Y Yanagida T Miyasaka M Engagement of CD44 promotes rac activation and CD44 eleavage during tumor cell migration J Biol Chem 2004 279 4541 4550 10.1074/jbc.M307356200 14623895

[B14] Lin B Hao YY Wang DD Zhu LC Zhang SL Saito M Iwamori M Transfection of α1,2-fucosyltransferase gene increase the antigenic expression of Lewis y in ovarian cancer cell line RMG-I Zhongguo Yi Xue Ke Xue Yuan Xue Bao 2008 30 284 289 18686606

[B15] Nonaka M Ma BY Murai R Nakamura N Baba M Kawasaki N Hodohara K Asano S Kawasaki T Glycosylation-dependent interactions of C-Type lectin DC-SIGN with colorectal tumor-associated lewis glycans impair the function and differentiation of monocyte-derived dendritic cells J Immunol 2008 180 3347 3356 18292560 10.4049/jimmunol.180.5.3347

[B16] Roseman S Reflections on glycobiology J Biol Chem 2001 276 41527 41542 10.1074/jbc.R100053200 11553646

[B17] Wang X Gu J Ihara H Miyoshi E Honke K Taniguchi N Core fucosylation regulates epidermal growth factor receptor-mediated intracellular signaling J Biol Chem 2006 281 2572 2577 10.1074/jbc.M510893200 16316986

[B18] Orczyk-Pawiłowicz M The role of fucosylation of glycoconjugates in health and disease Postepy Hig Med Dosw 2007 61 240 252 17507872

[B19] Baldus SE Hanisch FG Pütz C Flucke U Mönig SP Schneider PM Thiele J Hölscher AH Dienes HP Immunoreactivity of Lewis blood group and mucin peptide core antigens: correlations with grade of dysplasia and malignant transformation in the colorectal adenomaecarcinoma sequence Histol Histopathol 2002 17 191 198 11813869 10.14670/HH-17.191

[B20] Kiguchi K Iwamori M Mochizuki Y Kishikawa T Tsukazaki K Saga M Amemiya A Nozawa S Selection of human ovarian carcinoma cells with high dissemination potential by repeated passage of the cells in vivo into nude mice, and involvement of Le(x)-determinant in the dissemination potential Jpn J Cancer Res 1998 89 923 932 9818028 10.1111/j.1349-7006.1998.tb00650.x PMC5921947

[B21] Iwamori M Iwamori Y Kubushiro K Ishiwata I Kiguchi K Characteristic expression of Lewis-antigenic glycolipids in human ovarian carcinoma-derived cells with anticancer drug-resistance J Biol Chem 2007 141 309 317 10.1093/jb/mvm031 17190787

[B22] Zhu LC Lin B Hao YY Li FF Diao B Zhang SL Impact of α1,2-fucosyltransferase gene transfection on cancer-related gene expression profile of human ovarian cancer cell line RMG-I Ai Zheng 2008 27 934 941 18799031

[B23] Yue ZHAO Bei LIN Ying-Ying HAO Li-Mei YAN Juan-Juan LIU Lian-Cheng ZHU Shu-Lan ZHANG The effects of Lewis(y) antigenic content on drug resistance to Carboplatin in ovarian cancer line RMG-I Prog Biochem Biophys 2008 35 1175 1182

[B24] Juan-juan LIU Bei LIN Yue QI Fei-fei LI Ying-ying HAO Da-wo LIU Yue ZHAO Fan ZHANG Lian-cheng ZHU Shu-lan ZHANG Inhibitory effect of α-L-fucosidase on Lewis y antigen overexpressed human ovarian cancer cells in vitro J China Med Univ 2010 39 321 324

[B25] Lin WM Karsten U Goletz S Cheng RC Cao Y Co-expression of CD173 (H2) and CD174 (Lewis Y) with CD44 suggests that fucosylated histo-blood group antigens are markers of breast cancer-initiating cells Virchows Arch 2010 456 403 409 10.1007/s00428-010-0897-5 20300773

[B26] Yuan K Listinsky CM Singh RK Listinsky JJ Siegal GP Cell Surface Associated Alpha-L-Fucose Moieties Modulate Human Breast Cancer Neoplastic Progression Pathol Oncol Res 2008 14 145 156 10.1007/s12253-008-9036-x 18553163

[B27] Labarrière N Piau JP Otry C Denis M Lustenberger P Meflah K Le Pendu J H Blood Group Antigen Carried by CD44V Modulates Tumorigenicity of Rat Colon Carcinoma Cells J Cancer Res 1994 54 6275 6281 7525057

[B28] Bourguignon LY Singleton PA Zhu H Diedrich F Hyaluronan-mediated CD44 interaction with Rho GEF and Rho kinase promotes Grb2-associated binder-1 phosphorylation and phosphatidylinositol 3-kinase signaling leading to cytokine (macrophage-colony stimulating factor) production and breast tumor progression J Biol Chem 2003 278 29420 29434 10.1074/jbc.M301885200 12748184

[B29] Bourguignon LY Zhu H Shao L Chen YW CD44 Interaction with c-Src Kinase Promotes Cortactin-mediated Cytoskeleton Function and Hyaluronic Acid-dependent Ovarian Tumor Cell Migration J Biol Chem 2001 276 7327 7336 10.1074/jbc.M006498200 11084024

[B30] Liu J Lin B Hao Y Qi Y Zhu L Li F Liu D Cong J Zhang S Iwamori M Lewis y antigen promotes the proliferation of ovarian carcinoma-derived RMG-I cells through the PI3K/Akt signaling pathway J Exp Clin Cancer Res 2009 28 154 165 10.1186/1756-9966-28-154 20003467 PMC2806302

[B31] Gardner MJ Jones LM Catterall JB Turner GA Expression of cell adhesion molecules on ovarian tumour cell lines and mesothelial cells, in relation to ovarian cancer metastasis Cancer Lett 1995 91 229 234 10.1016/0304-3835(95)03743-G 7539337

[B32] Kaneko O Gong L Zhang J Hansen JK Hassan R Lee B Ho M Binding Domain on Mesothelin for CA125/MUC16 J Biol Chem 2009 284 3739 3749 10.1074/jbc.M806776200 19075018 PMC2635045

[B33] Makrydimas G Zagorianakou N Zagorianakou P Agnantis NJ CD44 family and gynaecological cancer In Vivo 2003 17 633 640 14758731

[B34] Pure E Cytokines regulate the affinity of solube CD44 for hyaluronan FEBS Lett 2004 556 69 74 10.1016/S0014-5793(03)01370-X 14706828

[B35] Fujisaki T Tanaka Y Fujii K Mine S Saito K Yamada S Yamashita U Irimura T Eto S CD44 stimulation induces integrin-mediated adhesion of colon cancer cell lines to endothelial cells by up-regulation of integrins and c-Met and activation of integrins J Cancer Res 1999 59 4427 4434 10485493

[B36] Wielenga VJ van der Voort R Taher TE Smit L Beuling EA van Krimpen C Spaargaren M Pals ST Expression of c-Met and heparan-sulfate proteoglycan forms of CD44 in colorectal cancer Am J Pathol 2000 157 1563 1573 10.1016/S0002-9440(10)64793-1 11073815 PMC1885727

[B37] Zhang L Wang YW Lang SX Research of the signal pathway of CD44-HA in colorectal carcinoma China Med Engineering 2006 14 586 589

[B38] Kim HR Wheeler MA Wilson CM Iida J Eng D Simpson MA McCarthy JB Bullard KM Hyaluronan facilitates invasion of colon carcinoma cells in vitro via interaction with CD44 J Cancer Res 2004 64 4569 4576 10.1158/0008-5472.CAN-04-0202 15231668

[B39] Yan LM Lin B Zhu LC Hao YY Qi Y Wang CZ Gao S Liu SC Zhang SL Iwamori M Enhancement of the adhesive and spreading potentials of ovarian carcinoma RMG-1 cells due to increased expression of integrin alpha5beta1 with the Lewis Y-structure on transfection of the alpha1,2-fucosyltransferase gene Biochimie 2010 92 852 857 10.1016/j.biochi.2010.02.012 20172014

[B40] Liu JJ Lin B Hao YY Li FF Liu DW Qi Y Zhu LC Zhang SL Iwamori M Lewis(y) antigen stimulates the growth of ovarian cancer cells via regulation of the epidermal growth factor receptor pathway Oncol Rep 2010 23 833 841 10.3892/or_00000730 20127027

